# Superior Strength-Ductility Synergy Enabled by Dual-Level Heterostructure of L1_2_ Precipitates and Local Chemical Order in a MPEA

**DOI:** 10.3390/nano16070418

**Published:** 2026-03-30

**Authors:** Jingjing Zhang, Yongfeng Shen, Wenying Xue, Zhijian Fan

**Affiliations:** 1 Key Laboratory for Anisotropy and Texture of Materials (Ministry of Education), School of Materials Science and Engineering, Northeastern University, Shenyang 110819, China; 2 State Key Laboratory of Digital Steel, Northeastern University, Shenyang 110819, China; 3 Key Laboratory of Neutron Physics and Institute of Nuclear Physics and Chemistry, China Academy of Engineering Physics, Mianyang 621999, China

**Keywords:** multi-principal element alloy, L1_2_ Precipitates, local chemical order, strengthening, mechanical property

## Abstract

The trade-off between strength and ductility remains a pivotal challenge in the development of multi-principal element alloys (MPEAs) for structural applications. Here, we report a dual-scale ordering strategy to achieve triple strengthening in a Ni-26.6Co-18.4Cr-5.4Nb-4.1Mo-2.3Al-0.3Ti-0.05Y (wt.%) MPEA through the synergistic interplay of L1_2_ nanoprecipitates and local chemical order (LCO). The alloy was processed via cold rolling followed by aging at 750 °C for 8 h, resulting in a high density of coherent L1_2_ precipitates (average size 47 ± 1 nm, volume fraction ~27%) with an ultra-low lattice misfit of 0.5%. Additionally, sub-nanoscale LCO domains with an average diameter of 0.62 nm were identified within the face-centered cubic matrix. This hierarchical microstructure yields an exceptional combination of mechanical properties at room temperature: yield strength of 1480 ± 6 MPa, ultimate tensile strength of 1678 ± 10 MPa, and a total elongation of 13.9 ± 0.2%. Quantitative strengthening analysis reveals that precipitation strengthening (697 MPa) is the dominant contributor, followed by dislocation strengthening (397 MPa). Transmission electron microscopy characterization of deformed samples reveals that the low stacking fault energy, promoted by LCO, facilitates the dissociation of perfect dislocations and the formation of extensive stacking faults. The intersection of stacking faults on different {111} planes generates a large number of Lomer–Cottrell locks, which significantly enhance work hardening and delay plastic instability. The findings demonstrate that engineering dual-scale ordered structures offers a promising pathway for developing MPEAs with a superior strength-ductility combination.

## 1. Introduction

Multi-principal element alloys (MPEAs) utilize high configurational entropy to broaden the possibilities of alloy design, thereby providing more strategies to surmount the dilemma of the trade-off between strength and ductility. By incorporating multiple principal elements in near-equiatomic or non-equiatomic ratios, these alloys unlock an extensive and largely uncharted compositional space, offering unprecedented opportunities for microstructure control and property tailoring [1,2]. Consequently, MPEAs have emerged as a research frontier in physical metallurgy, renowned for their exceptional combination of mechanical properties, thermal stability, and corrosion resistance, which render them particularly attractive for service in extreme environments [3,4].

Face-centered cubic (FCC) systems, represented by CoCrNi alloys and their derivatives, have been widely recognized as promising candidates for engineering applications [5]. The high ductility, outstanding work-hardening capacity, and excellent low-temperature fracture toughness make them standout performers [6,7,8]. However, a critical and intrinsic limitation of FCC MPEAs is the relatively low yield strength at ambient temperatures [9]. This shortcoming, rooted in the inherent inclination of dislocation glide within the FCC lattice due to its low Peierls stress, poses a significant barrier to their deployment as high-strength structural materials [10].

Fortunately, precipitation strengthening has currently emerged as a potent strategy to further boost the strength of FCC MPEAs [11,12,13]. Among various precipitate phases, the L1_2_-ordered phase is particularly attractive due to its coherency with the FCC matrix, low interfacial energy, and excellent thermal stability, rendering it an ideal candidate for precipitation-strengthened MPEAs. For instance, Zhang et al. [14] recently demonstrated a breakthrough in alloy design by employing a CALPHAD-assisted approach to introduce a high density of L1_2_ nanoprecipitates (with a volume fraction of ~40% and an average size of ~20 nm) in a Co_40_Ni_30_Cr_20_Al_5_Ti_5_ alloy. This microstructure yielded an exceptional synergy of yield strength (1200 MPa) and ductility (35%) at −196 °C. The underlying mechanism was attributed to the coupling between low stacking fault energy (SFE) and the coherent nanoprecipitates. In addition, Lei et al. [15] developed a long-range ordered MPEA (Ni_38.6_Co_20.2_Fe_20.3_V_20.9,_ in at %), achieving an ultrahigh tensile strength exceeding 1600 MPa and an outstanding ductility of 30% at room temperature. The simultaneous activation of unusual dislocation multiple slip and stacking faults (SFs) in the κ phase, along with nano-SF networks, Lomer–Cottrell (L-C) locks, and high-density dislocations in the coupled L1_2_ and FCC phases, contributed to enhanced strain hardening and excellent ductility.

Additionally, local chemical order (LCO), an atomic-scale structural feature inherent to MPEAs, has recently emerged as a research frontier in the field [16,17]. Unlike long-range ordered precipitates, LCO manifests as sub-nanoscale compositional fluctuations or short-range order (SRO), typically spanning only a few atomic layers (less than 1 nm in size) [18]. As reviewed by Liu et al. [19], the formation of LCO is governed by the interplay of thermodynamic driving forces and kinetic constraints, yet its precise influence on mechanical behavior remains a subject of ongoing debate. On one hand, LCO can act as short-range obstacles to dislocation motion, impeding glide and contributing to strengthening by modulating the energy landscape of the slip plane. On the other hand, pronounced LCO may promote planar slip localization, potentially leading to premature failure and reduced ductility. A recent study has added a new layer of complexity to this understanding [20]. Investigating a high-entropy alloy subjected to intermediate-temperature aging, the researchers observed an intriguing “ductile-brittle-ductile” transition. Notably, the embrittlement was not attributable to conventional grain boundary weakening but rather originated from the synergistic interplay between the over-ordering of L1_2_ precipitates and the formation of serrated phase boundaries. This finding underscores the intricate coupling between ordered structures at different length scales (from atomic-scale LCO to nanoscale L1_2_ precipitates) and their collective impact on phase boundary characteristics and mechanical performance.

The formation of stacking faults and L-C locks is a crucial deformation mechanism in FCC structural materials [21,22]. Low SFE promotes the dissociation of perfect dislocations into Shockley partial dislocations, giving rise to extended stacking faults [23]. When stacking faults on different slip planes intersect, the leading partial dislocations can react to form immobile L-C locks, which act as effective barriers to subsequent dislocation motion, thereby enhancing work hardening [24]. A recent study by Stumpf et al. [25] has revealed an intriguing avenue for tailoring this deformation behavior. By introducing trace amounts of 0.4 at.% carbon into a CrCoNi alloy, they observed a significant suppression of the transformation-induced plasticity (TRIP) and twinning-induced plasticity (TWIP) effects. Instead, the alloy exhibited a high density of stacking fault networks and L-C locks, leading to a remarkable increase in hardness, from an initial value of 7.3 GPa, corresponding to a 22% enhancement. This finding demonstrates the feasibility of actively engineering dislocation configurations through compositional modulation of SFE, offering a new paradigm for optimizing mechanical properties beyond conventional deformation mechanisms.

This study explores the microstructural evolution of an MPEA with a focus on the precipitation behavior of the L1_2_ phase. The size distribution, crystallographic orientation, interface misfit, and atomic-scale features of the L1_2_ precipitates were quantitatively analyzed. Correlating these microstructural observations with room-temperature tensile properties and fracture morphology, the critical role of the dual-scale precipitation structure in modulating dislocation behavior was elucidated. A key finding is the pronounced formation of L-C locks, which can be identified as a dominant contributor to the enhanced work hardening behavior. The interaction between nanoscale L1_2_ precipitates and sub-nanoscale LCO is found to promote the dissociation of perfect dislocations and the subsequent formation of these immobile locks, thereby sustaining a high work hardening rate and delaying plastic instability. These insights not only advance the fundamental understanding of deformation mechanisms in precipitation-strengthened MPEAs but also provide a practical microstructural design strategy for developing novel alloys that simultaneously exhibit ultra-high strength and excellent ductility.

## 2. Materials and Methods

An MPEA was fabricated via vacuum arc melting and re-melted at least six times to ensure chemical homogeneity. The actual chemical composition of the as-cast ingot, measured by inductively coupled plasma, was Ni-26.6Co-18.4Cr-5.4Nb-4.1Mo-2.3Al-0.3Ti-0.05Y (wt.%). The ingot was then sectioned into a billet with dimensions of 60 mm × 30 mm × 8 mm via wire electrical discharge machining, followed by a homogenization heat treatment at 1200 °C for 4 h in an argon atmosphere. The material after the solid solution was named ST. Subsequently, the homogenized billet was cold-rolled to reduce its thickness from 8 mm to 2 mm. The resultant plate was hereinafter referred to as CR. Finally, a stress-relief annealing was conducted at 950 °C for 1 h. To investigate the precipitates’ evolution, aging treatments were performed at 750 °C for 8 h, and the corresponding aged specimen was designated as A-8.

Microstructural characterization was performed on a scanning electron microscope (SEM, FEI Apreo 2S, FEI, Lausanne, Switzerland) and a transmission electron microscope (TEM, JEOL JEM-2100F, JEOL Ltd., Tokyo, Japan). For SEM analysis, samples were mechanically ground and polished, followed by electrolytic polishing using an electrolyte of 10% perchloric acid in ethanol at 20 V for 30 s. EBSD maps were acquired at an accelerating voltage of 20 kV with a step size of 60 nm and processed using the AZtecCrystal software (Version 2.1). Thin foils (3 mm in diameter) for TEM observations were prepared by mechanical grinding to ~50 μm thickness, followed by twin-jet electrophishing (Tenupol-5, Thermo Fisher Scientific, Waltham, MA, USA) in a solution of 10% perchloric acid in methanol at 20 V and −25 °C. Subsequently, TEM observations were conducted at an acceleration voltage of 200 kV.

Uniaxial tensile tests were conducted on a universal testing machine (Shimadzu (Kyoto, Japan), AG-Xplus 100 kN) at room temperature under a constant strain rate of 1 × 10^−3^ s^−1^. Dog-bone-shaped specimens were fabricated along the rolling direction via wire electrical discharge machining. The gauge section of the specimens had dimensions of 20 mm in length, 5 mm in width, and 1.5 mm in thickness. To assess the temperature-dependent tensile properties and ensure data reliability, a minimum of three parallel tests were performed for each condition.

## 3. Results

### 3.1. Initial Microstructures

**Figure 1:**
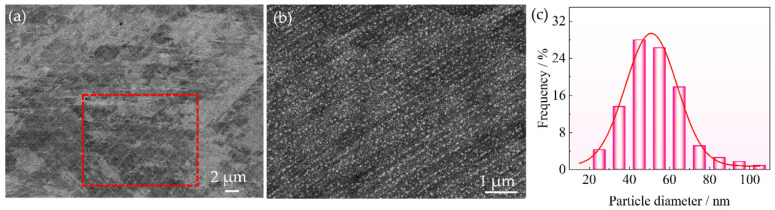
SEM image (**a**), the corresponding magnification of the rectangle (**b**), and precipitate size distribution histograms (**c**) of the A-8 sample.

Figure 1 presents the SEM micrographs and corresponding particle size distributions of the A-8 sample. No recrystallized grains with clear grain boundaries can be seen (Figure 1a) due to the annealing temperature below the recrystallization temperature of the alloys [26,27]. Instead, the microstructure is characterized by deformation bands introduced by rolling, which remain clearly visible throughout the sample, characterized by the parallel lines and intersecting lines. Impressively, a high density of spherical nanosized precipitates can be observed in the A-8 sample (Figure 1b). These fine precipitates are preferentially distributed along the shear bands, exhibiting a white-lined configuration. This distribution pattern is attributed to the high deformation energy storage and elevated dislocation density within the shear bands, which provide favorable nucleation sites and enhanced diffusion kinetics for precipitation during aging. Similarly, Jang et al. [28] proposed a shear band-driven dispersion of nanosized and semi-coherent precipitates, which exhibited significant strengthening effects. Micro-shear bands act as heterogeneous nucleation sites and generate finely dispersed intragranular precipitates with a semi-coherent interface, thereby leading to a remarkable strength-ductility balance. Furthermore, based on statistical analysis from 20 SEM images (total > 3000 particles for each condition), the mean particle diameter is determined to be 47 ± 1 nm in the A-8 sample, as shown in Figure 1c.

**Figure 2:**
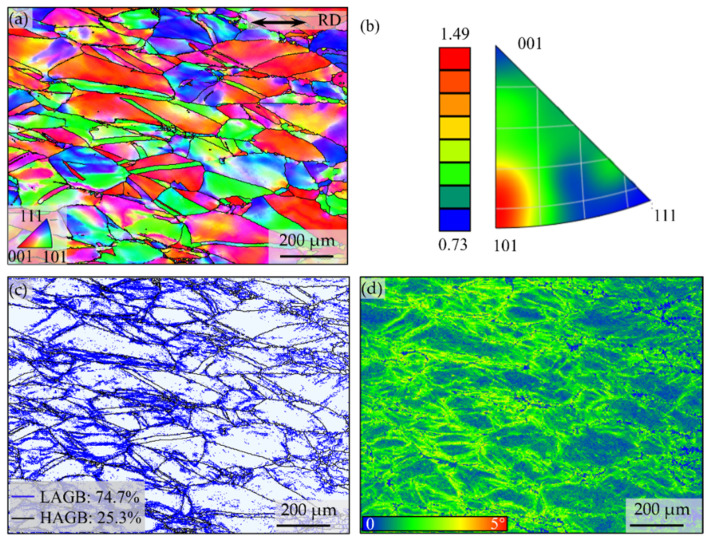
Orientation distribution map (**a**), inverse pole figure (**b**), the corresponding grain boundaries distribution (**c**), and local KAM (**d**) maps of the A-8 sample.

The EBSD characterizations of the A-8 sample are presented in Figure 2. The inverse pole figure (IPF) reveals the crystallographic orientation of the grains (Figure 2a). The corresponding texture analysis (Figure 2b) indicates that the sample has a clear preferential orientation, with a preference for grains oriented with {101} planes parallel to the rolling direction ({101} // RD). The grain boundary distribution map is shown in Figure 2c, where low-angle grain boundaries (LAGBs, with misorientation angles between 2° and 15°) and high-angle grain boundaries (HAGBs, with misorientation angles greater than 15°) are delineated in black and blue, respectively. Quantitative analysis reveals that LAGBs constitute a substantial fraction of 74.7% of the total grain boundaries, while HAGBs account for the remaining 25.3%. The high proportion of LAGBs is a typical characteristic of a deformed or recovered microstructure, suggesting that the sample did not undergo significant recrystallization [29]. From a perspective of strengthening, such a dense network of LAGBs is favorable as they act as effective obstacles to dislocation motion, thereby contributing to the high yield strength of the material. Local plastic strain distribution is provided by the Kernel Average Misorientation (KAM) map in Figure 2d. The average KAM value for the scanned area is 1.34°. This relatively moderate KAM value indicates dense geometrically necessary dislocations (GNDs) were accumulated during deformation, supported by the blurred areas near the grain boundaries. Notably, despite the dominance of LAGBs indicative of an unrecrystallized state, the KAM value is not excessively high. This could imply that dynamic recovery processes have occurred during heat treatment, rearranging dislocations into lower-energy configurations (i.e., LAGBs) and reducing the overall intragranular misorientation. The combination of a strong {101} // RD texture, a high LAGB fraction, and a moderate KAM value collectively suggests a microstructure that has undergone deformation and subsequent recovery without static recrystallization.

**Figure 3:**
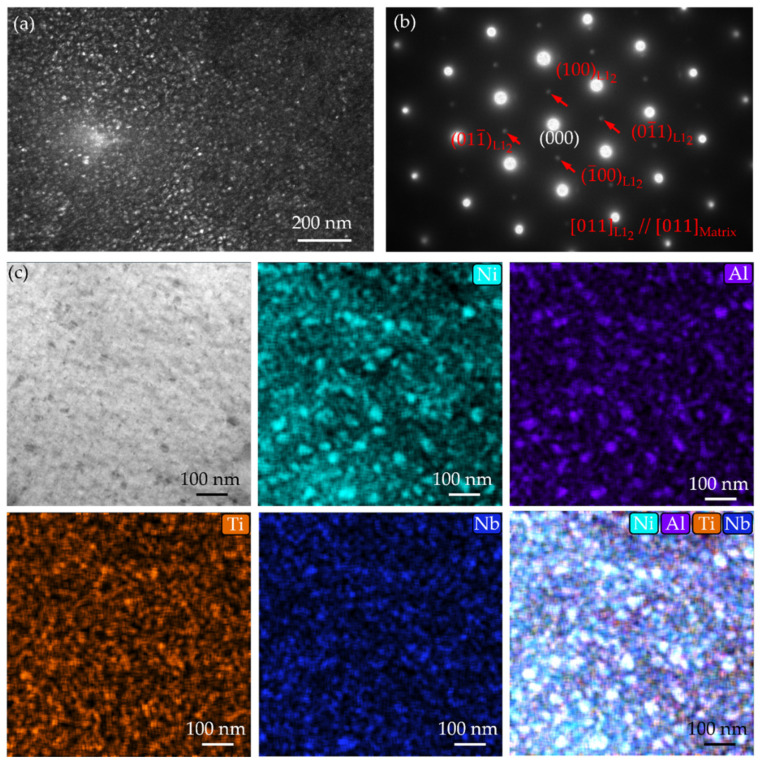
TEM dark-field image (**a**), corresponding SAED pattern (**b**), TEM image (**c**), and the corresponding EDS mapping of the A-8 sample.

To determine the features of precipitates in the MPEAs, elaborate transmission electron microscopy (TEM) characterizations were conducted. Figure 3 presents the TEM dark-field image, the corresponding selected area electron diffraction (SAED) pattern, and energy dispersive spectroscopy (EDS) mapping of the A-8 sample. As shown in Figure 3a, dense nanoscale precipitates with spherical morphology homogeneously distribute in the face-centered cubic (FCC) matrix. The dark-field imaging condition was set using a superlattice reflection to maximize the contrast of the ordered phase. The corresponding SAED pattern taken along the [011] zone axis (Figure 3b) reveals not only the fundamental spots from the disordered matrix but also distinct superlattice reflections (red arrows). The presence of these characteristic superlattice spots provides definitive evidence for the existence of an ordered L1_2_ structure. Furthermore, the perfect alignment of these superlattice spots with the matrix fundamental spots confirms a coherent orientation relationship between the two phases: [011]_L12_//[011]_matrix_. The compositions of these precipitates were elucidated by the EDS elemental maps (Figure 3c). The maps clearly demonstrate that the spherical particles are significantly enriched in Ni, Al, and Ti. Based on these results, the precipitates can be identified as the Ni_3_(Al, Ti, Nb) phase.

**Figure 4:**
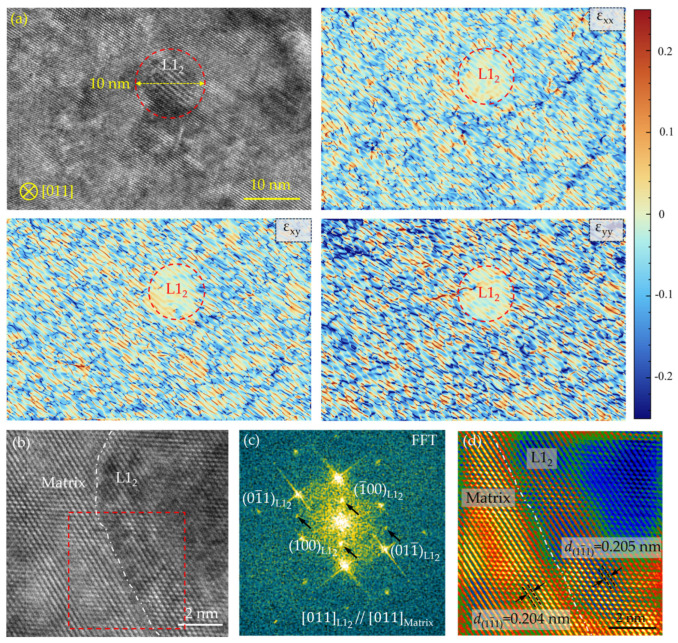
HRTEM image and corresponding GPA maps (**a**), HRTEM image showing the interface between matrix and L1_2_ phase (**b**), corresponding FFT (**c**), and IFFT patterns (**d**) of A-8 sample.

To further elucidate the microstructure of sample A-8, TEM analysis was performed. Figure 4a presents a high-resolution TEM (HRTEM) image of the A-8 sample along with its corresponding strain map obtained from geometric phase analysis (GPA). A spherical L1_2_ particle with a diameter of 10 nm can be clearly distinguished, and there is a highly localized strain field in the matrix region, whereas the interior of the L1_2_ precipitates exhibits a notably low strain state. This phenomenon can be attributed to the high-density dislocations retained in the matrix after the rolling-annealing processing, which introduce significant lattice distortion. During subsequent aging, the precipitation of the L1_2_ phase facilitates the annihilation and rearrangement of dislocations in the surrounding matrix, thereby locally relieving the lattice strain. Furthermore, the coherent nature of the L1_2_ precipitates with the matrix, combined with their low internal defect density, contributes to the minimal strain observed within the precipitates.

In addition, HRTEM was employed to examine the interface between the matrix and the L1_2_ precipitates, as shown in Figure 4b, showing a clear interface between the matrix and the L1_2_ phase. A Fast Fourier Transform (FFT) pattern obtained from the region within the square (Figure 4c) reveals distinct superlattice reflections. Indexing of this pattern confirms that the crystallographic orientation relationship between the two phases follows [011]_L12_//[011]_matrix_. To quantify the lattice mismatch at the interface, the interplanar spacings of the $\left\{111\right\}$ planes were measured. Using the corresponding Inverse Fast Fourier Transform (IFFT) image (Figure 4d), the measured d-spacings are approximately 0.204 nm for the FCC matrix and 0.205 nm for the L1_2_ phase. Based on these values, the lattice misfit (δ) is calculated to be about 0.005 (0.5%). This minimal misfit, combined with the continuous lattice fringes observed across the interface in the HRTEM image (Figure 4b), provides strong evidence for a coherent interface between the L1_2_ precipitate and the matrix.

**Figure 5:**
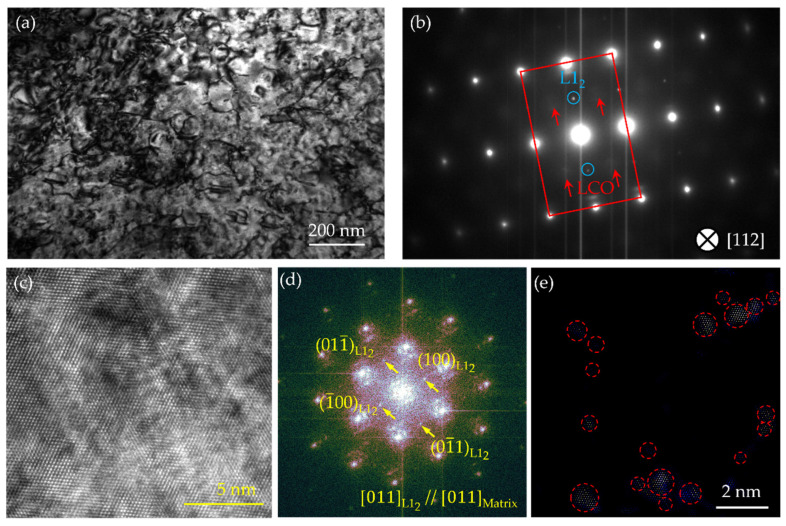
TEM picture (**a**), the corresponding SAED pattern (**b**), HRTEM image (**c**), and FFT (**d**) and IFFT patterns (**e**) of the A-8 sample. The red rectangle and blue circle in (**b**) represent the spots of the matrix and the superlattice diffraction spots of L1_2_ precipitates, respectively, and the red circle in (**e**) represents the LCO domains.

Figure 5a presents a bright-field TEM image of the A-8 sample, revealing a significant dislocation accumulation within the matrix. The corresponding SAED pattern taken along the [112] zone axis (Figure 5b) displays sharp extra superlattice reflections at 1/2{311} positions (indicated by red arrows), in addition to the fundamental FCC spots. These reflections are consistent with the presence of local chemical ordering (LCO) [30]. Figure 5c shows an HRTEM image of the A-8 sample viewed along the [011] axis. The corresponding FFT pattern (inset of Figure 5c) reveals reflections characteristic of the L1_2_ phase, indicating its widespread precipitation (Figure 5d). Notably, the interface between the L1_2_ precipitates and the matrix is coherent, with lattice fringes continuously traversing the boundary, consistent with the low lattice misfit calculated above. The FFT analysis further confirms that the orientation relationship obeys [011]_L12_//[011]_matrix_. To further investigate the LCO in detail, an IFFT image was generated by filtering the superlattice reflections. As shown in Figure 5e, the IFFT image reveals nanoscale atomic aggregations (highlighted by the red broken circles), corresponding to the LCO domains. Statistical analysis of over 30 such IFFT images yields an average diameter of approximately 0.6 ± 0.1 nm for these LCO domains, suggesting they consist of only several atomic layers.

### 3.2. Mechanical Properties of the MPEAs

**Figure 6:**
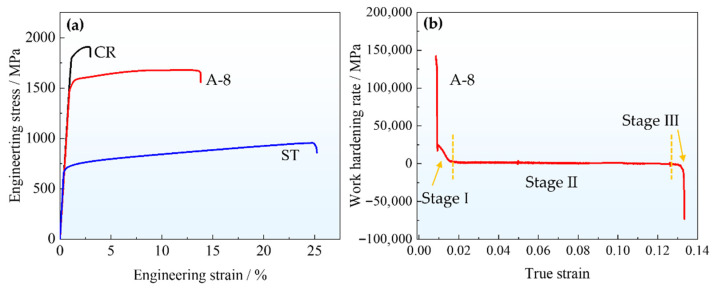
Engineering stress–strain curves of the samples under different processing (**a**), and work hardening rate curve of the A-8 sample (**b**).

The mechanical properties at room temperature of the MPEAs under various processing conditions were evaluated, with the engineering stress–strain curves and work hardening rate curve presented in Figure 6. The sample after solid solution treatment (ST) shows good processability, with a yield strength (YS) of 677 ± 8 MPa, an ultimate tensile strength (UTS) of 956 ± 8 MPa, and a total elongation (TE) of 24.7 ± 0.5% (Figure 6a). After cold rolling (CR), the strength of the sample is significantly improved, accompanied by a loss of ductility. Specifically, the YS of the CR sample is as high as 1796 ± 10 MPa, with UTS of 1907 ± 10 MPa and a TE of only 1.9 ± 0.2%. In contrast, the sample after aging treatment (A-8) shows a good combination of strength and ductility, with YS, UTS, and TE reaching 1480 ± 10 MPa, 1678 ± 10 MPa, and 13.9 ± 0.2%, respectively. This synergistic improvement can be attributed to the microstructural evolution during prolonged aging, where an increased volume fraction of coherent L1_2_ precipitates (Figure 1) provides enhanced precipitation strengthening.

As shown in Figure 6b, the A-8 sample exhibits a three-stage characteristic in its work-hardening rate curves. In Stage I, the work hardening rate decreases rapidly. This initial drop is attributed to the transition from elastic to plastic deformation, during which dislocations glide primarily on a few favorably oriented slip systems with relatively low resistance. When the strain increases, the multiplication of dislocations and the activation of additional slip systems lead to an increase in deformation resistance, resulting in the observed sharp decline in hardening rate. In Stage II, the work hardening rate stabilizes at a relatively low and nearly constant level, forming a plateau. This corresponds to the onset of multiple slip, where dislocations on intersecting slip systems interact, forming entanglements and substructures. The dynamic equilibrium between dislocation accumulation and annihilation maintains a stable resistance to deformation, thereby keeping the hardening rate approximately constant. In Stage III, the work hardening rate begins to decrease progressively and eventually becomes negative, marking the onset of necking. Once the hardening rate drops below zero, plastic instability occurs, leading to strain localization, accelerated deformation in the necked region, and eventual fracture.

## 4. Discussion

### 4.1. Derivation of High Yield Strength

To quantitatively evaluate the individual contributions of various strengthening mechanisms to the overall yield strength of the as-prepared MPEA, we systematically analyzed the strengthening effects arising from solid solution, grain boundaries, dislocations, and precipitates. The theoretical yield strength ($\sigma_{y}$) of the alloy can be expressed as a linear superposition of these individual contributions [31]:

(1) $$\sigma_{y}=\sigma_{0}+\sigma_{s}+\sigma_{d}+\sigma_{p}+\sigma_{g}+\sigma_{LCO}$$

here $\sigma_{y}$ is the yield strength of the material, $\sigma_{0}$ is lattice friction stress, while $\sigma_{s}$, $\sigma_{d}$, $\sigma_{p}$, $\sigma_{g}$ and $\sigma_{LCO}$ denote the contribution of solid-solution strengthening, dislocation strengthening, precipitation strengthening, grain refinement strengthening, and strengthening induced by LCO, respectively.

The Fleischer model predicts the solid solution strengthening in the MPEA through the following model [10]:

(2) $$\sigma_{s}=\frac{{{\mathrm{MG}\varepsilon }_{s}}^{\frac{3}{2}}c^{\frac{1}{2}}}{700}$$

where M = 3.06 is the Taylor factor, G = 77.2 GPa is the shear modulus of the matrix, c is the solute concentration in the molar fraction (3.6%), $\varepsilon_{s}$ is the solvent-solute interaction parameter,

(3) $$\varepsilon_{s}=|\frac{\eta_{\mathrm{em}}}{1+0.5\eta_{\mathrm{em}}}-3\eta_{\mathrm{am}}|$$

here $\eta_{\mathrm{em}}=\frac{1}{G}\frac{\mathrm{dG}}{\mathrm{dc}}$ and $\eta_{\mathrm{am}}=\frac{1}{a}\frac{da}{\mathrm{dc}}$ is the elastic modulus misfit and the atomic size misfit, respectively. Due to similar atomic radii, Ni, Co, and Cr are selected as the solvent atoms, while the remaining elements serve as solute atoms. Thus, the $\sigma_{s}$ of the A-8 sample is determined to be 104 MPa.

The grain boundary strengthening is commonly calculated by the classical Hall-Petch relationship. Due to the absence of recrystallized grains in the material used in this experiment, grain strengthening can be neglected. Dislocation strengthening can be estimated by the following equation:

(4) $$\sigma_{d}=\alpha \mathrm{MG}b\sqrt{\rho_{\mathrm{GND}}}$$

here α is a constant related to the nature of dislocations (0.2), and $\rho_{\mathrm{GND}}$ is the density of geometrical necessary dislocations (GNDs, 1.11 × 10^−21^ m^−2^). Thus, the $\sigma_{d}$ of A-8 sample is 397 MPa.

Meanwhile, the contribution of L1_2_ precipitates in the A-8 sample to yield strength can be quantified by the larger one of $\Delta \sigma_{\mathrm{CS}}$ + $\Delta \sigma_{\mathrm{MS}}$ and $\Delta \sigma_{\mathrm{OS}}$ [31]:

(5) $$\Delta \sigma_{\mathrm{CS}}=M\alpha_{\varepsilon }{\left(G\cdot \varepsilon \right)}^{\frac{3}{2}}{\left(\frac{rf}{0.5Gb}\right)}^{\frac{1}{2}}$$

(6) $$\Delta \sigma_{\mathrm{MS}}=0.0055M{\left(\Delta G\right)}^{\frac{3}{2}}{\left(\frac{2f}{G}\right)}^{\frac{1}{2}}{\left(\frac{r}{b}\right)}^{\frac{3}{2}m-1}$$

(7) $$\Delta \sigma_{\mathrm{OS}}=0.81M\frac{\gamma_{\mathrm{APB}}}{2b}{\left(\frac{3\pi f}{8}\right)}^{\frac{1}{2}}$$

here $\alpha_{\varepsilon }$ = 2.6 for FCC structure, M = 0.85, ε = 0.00326 in this study, ΔG = 10 GPa is the shear modulus mismatch between precipitates and matrix, γ_APB_ is defined as the energy associated with the antiphase domain boundary (APB) of the L1_2_ phase, quantified as 0.25 J/m^2^ [31]. In addition, precipitate dimensions are analyzed using Image-Pro Plus 6.0 software. The area fraction, obtained from 20 random regions, is taken as an approximation of the volume fraction following standard stereological principles. Consequently, the volume fraction (*f*) of the L1_2_ precipitates was measured to be 27%. Thus, the calculated $\Delta \sigma_{\mathrm{OS}}$ of A-8 sample is as high as 697 MPa, greater than the sum of $\Delta \sigma_{\mathrm{CS}}$ (23 MPa) and $\Delta \sigma_{\mathrm{MS}}$ (103 MPa), i.e., $\sigma_{p}$ is determined to be 697 MPa in the A-8 sample. Unlike conventional long-range ordered L1_2_ precipitates, which strengthen alloys via anti-phase boundary, local chemical order (LCO) enhances strength by modulating the energy landscape for dislocation glide. The LCO introduces localized fluctuations in the atomic bonding environment, creating short-range barriers that impede dislocation motion. Molecular dynamics simulations by Li et al. [32] on a NiCoCr alloy suggest that the presence of LCO can contribute an intrinsic shear strength increment of ~70 MPa, providing a quantitative reference for the potential strengthening contribution from this sub-nanoscale feature. Thus, the value of $\sigma_{LCO}$ in this study can be estimated to be 70 MPa based on the above research. Meanwhile, Zhang et al. [33] obtained a value of $\sigma_{0}$ = 293 MPa for the material with similar chemical compositions. The calculated $\sigma_{y}$ of 1561 MPa is close to the tested value. The increment of $\sigma_{y}$ triggered by the bimodal distribution of nano-precipitates is approximately 45%. Distinctly, precipitation strengthening is the pivotal strengthening factor in the A-8 sample.

### 4.2. Deformation Mechanisms of the MPEA

**Figure 7:**
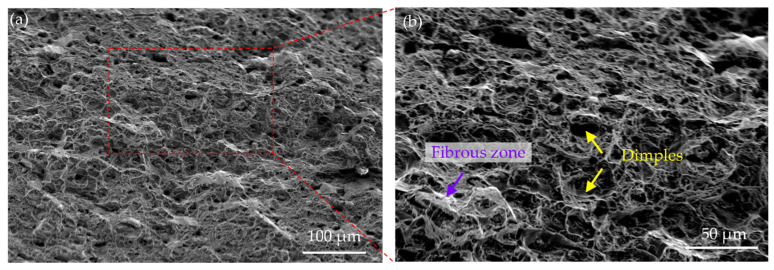
(**a**) SEM images showing fracture morphologies of the A-8 sample, (**b**) partial enlargement.

To analyze the correlation between the ductility and the microstructure of the MPEA, the morphology and the microstructure feature near the fracture surface were observed (Figure 7). It was observed that the fracture surface contains numerous dimples with varying sizes and depths (Figure 7a,b). These dimples were created by the significant plastic deformation of the material before fracture, where micropores nucleated, grew, and ultimately connected to inclusions or second-phase particles. The morphology of the ductile dimples is mainly slightly elongated, indicating the presence of both normal stress and shear stress during the fracture process. The deep dimples are connected by fiber zones, and the presence of these zones further confirms that the material underwent significant plastic deformation before fracture. In addition, the L1_2_ precipitates distributed along the rolling deformation zone in the A-8 sample are mostly nanoscale and coherent with the matrix, indicating good interface bonding, thereby preserving high strain compatibility and reducing stress concentration as well as microcrack initiation. During deformation, dislocations can cut through the small L1_2_ precipitates, causing the two phases to deform synergistically and avoid interface debonding, consequently promoting the plastic deformation. In particular, dense precipitates can promote more uniform dislocation slip, thus avoiding premature and local concentration of plastic deformation.

**Figure 8:**
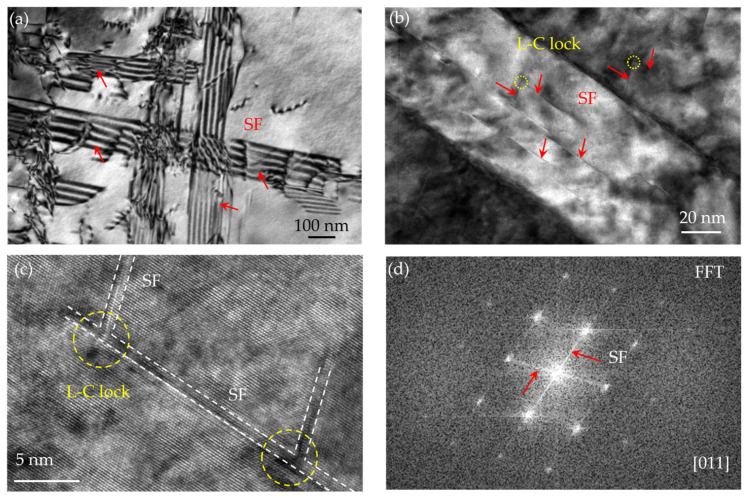
TEM observation of the A-8 sample deformed at room temperature: TEM images showing SFs (**a**) and L-C locks (**b**), the corresponding HRTEM image (**c**), and FFT pattern (**d**). The red arrow in (**d**) indicates the existence of SF.

Figure 8 shows the TEM characterization of the A-8 sample after tensile deformation. Profuse stacking faults (SFs) can be observed, tangled with a number of dislocations (Figure 8a). Close observation reveals the intersecting SFs on different {111} planes (Figure 8b). These SFs intersect at an angle of approximately 70.5°, which corresponds precisely to the angle between two {111} slip planes in a face-centered cubic (FCC) lattice, suggesting the formation of L-C locks. This configuration was frequently observed in the HRTEM image (Figure 8c). To confirm this, an FFT was performed on the region containing the intersecting SFs. As shown in Figure 8d, the streaks in the FFT pattern perpendicular to the SF planes confirm their planar nature. In particular, the two distinct streak directions correspond to two different {111} plane traces, providing definitive evidence for the formation of L-C locks at the intersection of these SFs [34]. The formation of numerous SFs is attributed to the addition of 26.6 wt.% Co, which decreases the SFE of the alloy. The SFE of the MPEA at room temperature was calculated as 1.6 mJ/m^2^ via JMatPro 15.0 software. A reduced SFE promotes the dissociation of perfect dislocations into Shockley partials bounding SFs, facilitating planar slip and contributing to improved ductility [23]. Crucially, these L-C locks act as potent barriers to incoming dislocations, causing them to pile up and generate significant back stresses. This activity not only enhances work hardening but also sets the stage for the activation of secondary slip systems, contributing to sustainable plastic deformation [35]. Thus, the dislocation storage capacity is significantly enhanced, and the work hardening rate is accordingly increased. It is well known that a higher work hardening rate is beneficial for delaying necking during tensile deformation, thereby improving the uniform elongation and overall strength-ductility synergy of the alloy.

**Figure 9:**
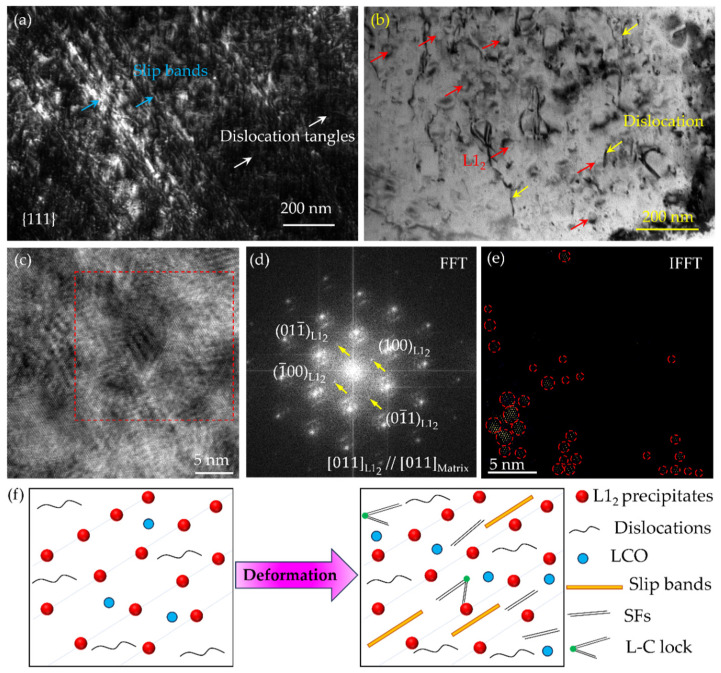
TEM images showing slip bands (**a**) and L1_2_ precipitates (**b**), HRTEM picture (**c**), and the corresponding FFT (**d**) of the red-box region in (**c**), along with IFFT patterns (**e**) of the square region. Schematic illustrations revealing the deformation mechanisms tested at room temperature (**f**). The blue and white arrows in (**a**) represent slip bands and dislocation tangles, respectively. The red and yellow arrows in (**b**) represent slip L1_2_ precipitates and dislocations, respectively.

Further insights into the deformation mechanisms of the A-8 sample are provided in Figure 9. A TEM image (Figure 9a) reveals abundant planar slip bands and pronounced dislocation pile-ups at slip band intersections and grain boundaries. The planar slip morphology observed on {111} planes is primarily attributed to the reduced SFE of the alloy. Low SFE promotes the dissociation of perfect dislocations into widely separated Shockley partials, which constrains dislocation motion to the original slip plane by suppressing cross-slip [36]. The observed dislocation pile-ups generate significant back stress, acting as effective obstacles to subsequent dislocation glide. Consequently, a higher applied stress is required to initiate further plastic flow, contributing directly to the enhanced yield strength and ultimate tensile strength of the materials. Furthermore, the uniform distribution of these slip bands during the early stages of tensile deformation promotes homogeneous strain distribution, which is beneficial for delaying strain localization and achieving a high uniform elongation.

In addition to dislocation substructures, dense dispersion of spherical L1_2_-ordered precipitates was observed (Figure 9b). Evidence of dislocation-precipitate interaction, specifically precipitate shearing by moving dislocations, is clearly distinguishable. The primary strengthening mechanisms in this regime arise from the coherent nature of these precipitates. The lattice misfit between the L1_2_ precipitates and the FCC matrix generates coherent strain fields that interact with the stress fields of gliding dislocations. Moreover, when a dislocation shears an ordered L1_2_ precipitate, it creates an APB on the slip plane, which substantially increases the energy required for dislocation motion [37,38]. This ordered strengthening effect, combined with the coherency strain, significantly elevates the resistance to dislocation glide. Once dislocations cut through these precipitates, they not only encounter the increasing resistance but also the mean free path for dislocation motion is effectively reduced. This promotes more rapid dislocation multiplication and accumulation, thereby further enhancing the work hardening rate and contributing to an excellent combination of high strength and good ductility. HRTEM was employed to further investigate the precipitate structure. Figure 9c presents an HRTEM image of a typical L1_2_ precipitate embedded in the FCC matrix. FFT analysis was performed on the region marked by the red dashed square (Figure 9c), and the resultant pattern (Figure 9d) clearly exhibits superlattice reflections at {110} positions. This confirms the L1_2_ ordered structure of the precipitates and demonstrates an orientation relationship with the disordered FCC matrix: [011]_L12_//[011]_matrix_. To gain deeper insight into the local lattice characteristics, an IFFT was conducted on the identical region. The IFFT image reveals localized lattice distortions or nanoscale strain fields in the vicinity of the precipitate-matrix interface (Figure 9e). Quantitative analysis indicates that these strained regions have a characteristic size of approximately 0.93 ± 0.2 nm, which has increased compared to those in the as-prepared sample. These nanoscale units are attributed to the coherent strains arising from the lattice misfit between the precipitate and the matrix. Crucially, such local strain fields act as additional short-range obstacles to dislocation motion. When gliding dislocations encounter these strained regions during deformation, they experience a fluctuating stress field that increases the resistance to dislocation propagation. This contributes to the overall precipitation strengthening beyond the classical order strengthening mechanism, particularly during the early stages of precipitate shearing. The presence of these nanoscale strain heterogeneities further refines the effective slip distance for dislocations, thereby augmenting the work hardening capacity and promoting a favorable strength-ductility synergy in the alloy.

Figure 9f presents the schematic illustrations showing the deformation mechanisms of the as-prepared MPEA under tensile testing at room temperature. Prior to tensile deformation, the A-8 sample is characterized by a high density of L1_2_ nanoprecipitates (preferentially nucleated along rolling-induced shear bands) and the presence of LCO in the matrix. At this stage, precipitation strengthening and dislocation strengthening are the primary contributors to the material’s ultrahigh yield strength. Based on the TEM characterization of the deformed A-8 sample, the deformed microstructure consists of L1_2_ precipitates, LCO, slip bands, SFs, and L-C locks. During plastic deformation, a cascade of deformation mechanisms is activated, collectively enhancing the work hardening capability. Specifically, the formation of extended stacking faults and the subsequent development of L-C locks act as effective barriers to dislocation motion, promoting dislocation pile-up and storage. Moreover, as discussed in the previous section, stress concentrations around these locks can activate adjacent dislocation sources, sustaining continuous dislocation generation. This dynamic interplay between dislocation blocking and regeneration underpins the continuous work hardening of the A-8 sample, enabling the exceptional combination of high strength and good ductility.

**Figure 10:**
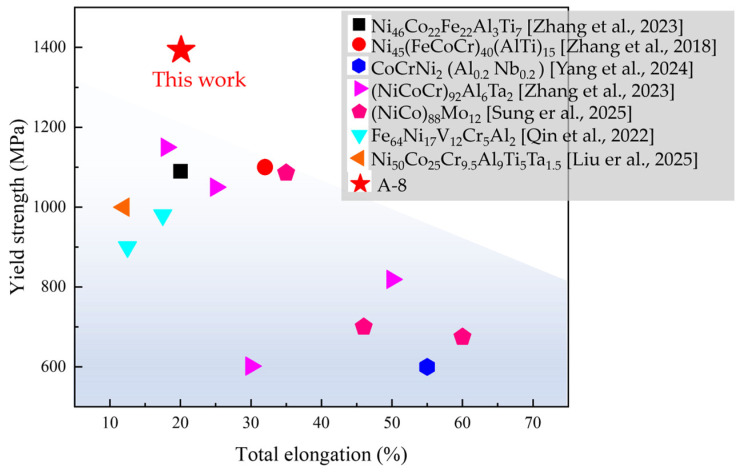
Mechanical properties of the A-8 sample compared with the reported data [39,40,41,42,43,44,45].

The superior combination of strength and ductility exhibited by the A-8 sample—yield strength of 1480 MPa, tensile strength of 1678 MPa, and elongation of 13.9%—can be attributed to the synergistic interplay among precipitation strengthening from dense L1_2_ nanoprecipitates, additional strengthening from LCO, and the activation of multiple deformation mechanisms such as the formation of L-C locks and stacking fault networks. This integrated strengthening/toughening strategy enables the A-8 alloy to outperform many recently reported high-performance materials [39,40,41,42,43,44,45], as shown in Figure 10. For instance, its yield strength is ~30% higher than that of a typical CoCrNi-based MPEA strengthened by L1_2_ precipitates (reported YS of ~1150 MPa [42]), while maintaining comparable ductility.

The excellent strength and plasticity of the alloy in this study are caused by multi-scale microstructure control. On the one hand, substantial nanoscale L1_2_ coherent precipitates (with a mismatch degree of only 0.5% with the matrix) effectively hinder dislocation movement through ordered strengthening and coherent strain strengthening. On the other hand, the LCO serves as a dispersed obstacle that impedes dislocation motion and significantly contributes to the yield strength. Under sufficiently high stress, dislocations cut through the LCO, which not only contributes to strengthening but also introduces local order disruption, temporarily lowering the region’s resistance to subsequent dislocations. This cutting-induced disordering establishes a dynamic equilibrium between softening and hardening at the microscopic level, preventing excessive dislocation pile-up in front of individual obstacles and thereby enabling continuous and stable deformation. Additionally, profuse SFs and L-C locks formed during the deformation process substantially enhance the work-hardening ability of the alloy. The microstructure analysis after deformation shows that the uniformly distributed plane slip bands and dislocation pile-up groups have effectively strengthened the back stress. Simultaneously, the size of LCO increased to about 0.93 nm after uniaxial tensile deformation, indicating that the locally ordered chemical structure interacted with dislocations during the deformation process, further enhancing the strengthening effect. The synergistic effect of the L1_2_ precipitation phase and LCO jointly promotes the combination of high strength and good plasticity of the alloy.

## 5. Conclusions

A novel multi-principal element alloy processed by cold rolling and heat treatment was developed, achieving a superior combination of strength and ductility. The main conclusions can be drawn as follows:

(1) The introduction of densely coherent L1_2_ nanoprecipitates with minimal lattice misfit (~0.5%) provides substantial precipitation strengthening, serving as the primary mechanism for the alloy’s ultra-high yield strength of 1480 ± 6 MPa.
(2) The presence of nanoscale local chemical order domains (~0.62 nm) reduces the stacking fault energy, thus promoting the dissociation of dislocations and the formation of extensive stacking faults during deformation.
(3) The interaction between stacking faults and different slip planes leads to the formation of numerous L-C locks, which act as effective barriers to dislocation motion, significantly enhancing the work hardening rate and delaying necking.
(4) The superior strength-ductility synergy is achieved through a dual-scale ordering strategy, where L1_2_ precipitates provide primary strengthening and local chemical order modulates dislocation glide, collectively activating multiple deformation mechanisms.

## Data Availability

The original contributions presented in this study are included in the article. Further inquiries can be directed to the corresponding author.
